# Impaired Maternal Behavior in *Usp46* Mutant Mice: A Model for Trans-Generational Transmission of Maternal Care

**DOI:** 10.1371/journal.pone.0136016

**Published:** 2015-08-18

**Authors:** Shoya Umemura, Saki Imai, Ayumi Mimura, Mari Fujiwara, Shizufumi Ebihara

**Affiliations:** Division of Biomodeling, Graduate School of Bioagricultural Sciences, Nagoya University, Nagoya, Japan; University of Rennes-1, FRANCE

## Abstract

*Usp46* mutant mice (congenic strain on a B6 genetic background; MT mice) have a low weaning rate and display poor maternal behavior compared to C57BL/6J mice (B6 mice). Based on these observations, we examined how maternal behavior is shaped by cross-fostering and in-fostering MT and B6 mice. The experiments consisted of six groups: B6 mice fostered by their biological mother (B6-CO); MT mice fostered by their biological mother (MT-CO); B6 mice fostered by a different B6 mother (B6-IF); MT mice fostered by a different MT mother (MT-IF); B6 mice fostered by an MT mother (B6-CF); and MT mice fostered by a B6 mother (MT-CF). Maternal behavior was assessed using the pup-retrieval test in adult female offspring, and four parameters, time nursing pups in the nest, time sniffing or licking pups, rearing behavior, and latency to retrieve pups, were measured. Cross-fostering significantly reduced time spent nursing and sniffing/licking pup, and increased the number of instances of rearing in the B6-CF group, and improved three parameters of maternal behaviors (nursing, rearing and latency) in the MT-CF group. These results indicate that the level of maternal care is transmitted to their pups and proper maternal behaviors can be shaped if adequate postpartum maternal care is given, even in genetically vulnerable mice. However, the offspring’s genotype may also influence the development of maternal behaviors in adulthood. Thus, MT mice may prove useful as a model for trans-generational transmission of maternal care, and these findings may provide insight into the mechanisms of maltreating behaviors in human child abuse.

## Introduction

Child abuse is prevalent worldwide and poses a serious public health risk because many, though not all, maltreated children are at risk of developing psychiatric problems (e.g. depression, anxiety, drug and alcohol abuse) in adulthood [1–5]. In Japan, the number of cases of child abuse handled in a consultation office for children has rapidly increased over the last 15 years, breaking a record for number of cases in 2013 (2013 Annual Report, Ministry of Health, Labor and Welfare, Japan). It has been reported that maltreating mothers experience more abuse and neglect during their own childhoods, relative to non-maltreating mothers [6]. This cycle of abuse has been described in the literature [7–10], but little is known of the physiological basis for this phenomenon. To better understand the mechanisms of this cycle, an appropriate animal model, that exhibits poor maternal behavior and a cycle of neglect similar to maltreating mothers, would be useful.

Recently, in a quantitative trait locus (QTL) genetic analysis using an F2 population cross between CS (an inbred strain originally established from a hybrid between NBC and SII in Nagoya University) and normal C57BL/6J (B6) mice, we identified ubiquitin specific peptidase 46 *(Usp46)* as one of the genes responsible for reductions in time spent immobile in the tail suspension and forced swim test [11]. The *Usp46* mutation identified in CS mice has a three base pair deletion coding for lysine in the open reading frame. Mice with this mutation (MT mice) exhibit a number of impaired behavioral phenotypes including nest building, ethanol preference, ethanol-induced loss of righting reflex, sucrose preference, novelty-suppressed feeding and marble burying behavior [11, 12]. It is thought that *Usp46* deficiency causes dysfunction of gamma-aminobutyric acid A (GABA_A_) receptor activity [11, 13]. Meanwhile, we noticed that the weaning rate of pregnant MT mice was lower than that of wild type mice. This observation suggested that maternal behavior in these mice might be altered. In fact, we found that maternal behavior during the first 3 days after birth was significantly impaired in MT mice. Many studies have suggested that both environmental factors during development and genetic factors are involved in the formation of maternal behavior [14–18]. Therefore, in this study we investigated how maternal behavior is shaped in MT and B6 mice by cross-fostering experiments.

## Materials and Methods

### Animals

We used B6 mice purchased from CLEA Japan Inc. and *Usp46* mutant mice developed as a congenic strain on a B6 genetic background. These congenic mice (B6.CS-Ngu1053) contained chromosome 5 regions harboring *Usp46* of CS mice (MT mice). In addition, we used *Usp46* transgenic mice, which were produced by introducing the wild type *Usp46* transgene to B6.CS-Ngu1053 mice. Mice were housed in polycarbonate cages (182 × 260 × 128 mm) with soft wooden chip (Chubu Kagaku Shizai) in our animal facility, under 12 h light/dark cycle (On 8:00, off 20:00), with free access to food (Labo MR Stock, NOSAN) and water, temperature and humidity maintained at approximately 24°C and 55%, respectively. In this study, we assessed maternal behavior in parous mice by video recording and nulliparous mice by a pup-retrieval test that were produced by the cross- and in-fostering procedure. To examine the number of pups in the uterus, mice were anesthetized with Somnopentyl. We performed the experiments in accordance with the Guidelines for Proper Conduct of Animal Experiments issued by Science Council of Japan (June 1, 2006) and the experiment was approved by the Animal Experiment Committee, Graduate School of Bioagricultural Sciences, Nagoya University (Approval No. 2011030303, 2012032101, 2013021808). The details of handling the mice are as follows.

1. Final cause of death for the animals: The mice used in this study were sacrificed by euthanasia procedures when the experiment was completed.
2. Humane endpoints: This study used humane endpoints.
  - The criteria to be humanely sacrificed: When disposing of mice on completion of the experiment in accordance with the animal experiment protocol or due to the laboratory animals being subjected to severe pain and suffering during the course of the experiment when anesthetics and analgesics could not be used, we conducted euthanasia.
  - The method of sacrifice: A chemical method (overdose of a barbiturate anesthetic) or a physical method (cervical dislocation) was used.
  - The frequency of monitor: Basically every day we checked water and food as well as health conditions.
  - To minimize suffering of the mice: To be able to perceive abnormalities, we checked physical conditions (body weight, hair etc.) and behavior (food intake, water consumption and activity etc.). If we perceived an abnormality and drugs (anesthetics, analgesics etc.) could not be used, the mice were sacrificed by euthanasia.

The completed ARRIVE checklist was attached as supplemental information (S2 Table).

### Observation of maternal behavior

Virgin B6 and MT female mice were crossed with male mice of the same strain; after pregnancy was confirmed, male mice were removed from the cage. A CCD (charge-coupled device) camera with night vision (Arucom, Japan) recorded maternal behavior (nursing position [arched-back or prone] and licking/grooming) for 3 days following parturition, at 30 min intervals (S1 Fig). During the record, mice were housed in a cage with wood-chip bedding. Some whole MT litters died during this period. In such a case, the observation of these mice was limited to only when the pup survived. The ratio of occurrence of maternal behaviors was recorded as the percent of total observation time. Recordings were stored in a DVD (digital versatile disc) recorder (Panasonic, Japan) or HDD (hard disk driver) with PCastVIDEO (Buffalo, Japan). Before analyzing the recording, the ratio of maternal behavior on the 4^th^ day following parturition was compared at three time intervals (10 s at 2, 3 and 6 min intervals during the 30 min recording). However, no significant differences were observed. Therefore, we analyzed the 30 min recording at 6 min intervals with 10 s assessments (5 points/h) (S1 Fig).

### Cross- and in-fostering procedure

**Fig 1:**
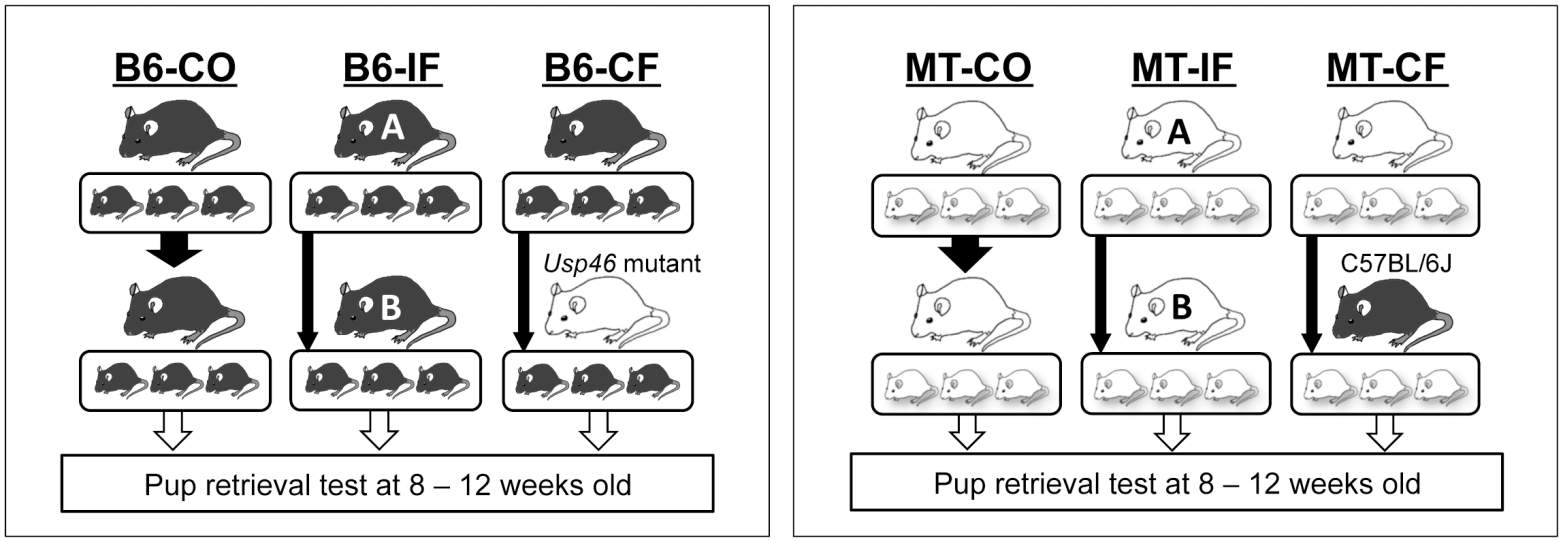
Outline of fostering experiments. B6-CO: B6 mice fostered by their biological mother. B6-IF: B6 mice fostered by a different B6 mother. B6-CF: B6 mice fostered by an MT mother. MT-CO: MT mice fostered by their biological mother. MT-IF: MT mice fostered by a different MT mother. MT-CF: MT mice fostered by a B6 mother. Offspring were tested at 8–12 weeks of age.

Virgin male and female B6 or MT mice (> 8 weeks of age) were crossed in each strain to obtain pregnant female mice. After confirming the pregnancy (at approximately 14 days of pregnancy), male mice were removed from the cage. Pups born from these mice were used in the pup-retrieval test after they grown up. The experiment consisted of six groups (Fig 1): B6 mice fostered by their biological mother (B6-CO); MT mice fostered by their biological mother (MT-CO); B6 mice fostered by a different B6 mother (B6-IF); MT mice fostered by a different MT mother (MT-IF); B6 mice fostered by an MT mother (B6-CF); and MT mice fostered by a B6 mother (MT-CF). Mothers for in-foster (IF) and cross-foster (CF) groups were exchanged only when litters were born within 2 days of each other. Pups were handled using tweezers during the short (< 5 min) exchange to avoid odors. Litters consisted of 4–9 pups, which were weaned at 25 days after birth. Male and female offspring (2–5 siblings in each cage) were separated at weaning and housed until the pup-retrieval test. In this test, only female mice (8–12 weeks old) were used.

### Pup-retrieval test

Test mice were isolated in a cage with wood-chip bedding and a nestlet made of pressed cotton (Lillico; 2.5 g, 5 × 5 cm) for 2 days. The experiment was performed on day 3 (10:00–12:00). B6 pups (1–5 days old), born from different B6 mothers, were placed in 3 corners of the cage (182 × 260 × 128 mm) and maternal behavior of the test mouse was recorded for 30 min using a video camera [19]. Four maternal behaviors were measured during playback: time spent nursing pups in the nest; time spent sniffing or licking each pup; number of instances of rearing (reflecting an interruption of nursing); and latency to retrieve each pup (1^st^, 2^nd^, 3^rd^) to the nest. In addition, we counted the number of pups in the nest at the end of the experiment and checked if infanticide occurred.

### Statistics

All data are expressed as the mean ± standard error of the mean (S.E.M). Statistical significance was determined using a number of different analyses including: one-way ANOVA with Tukey’s *post hoc* test; Kruskal-Wallis one-way ANOVA with Steel-Dwass *post hoc* test; two-way ANOVA with Tukey’s *post hoc* test for multiple comparisons; and Student’s *t*-tests or Mann-Whitney U test for 2 group comparisons.

## Results

### Effects of *Usp46* mutation on weaning rate

**Fig 2:**
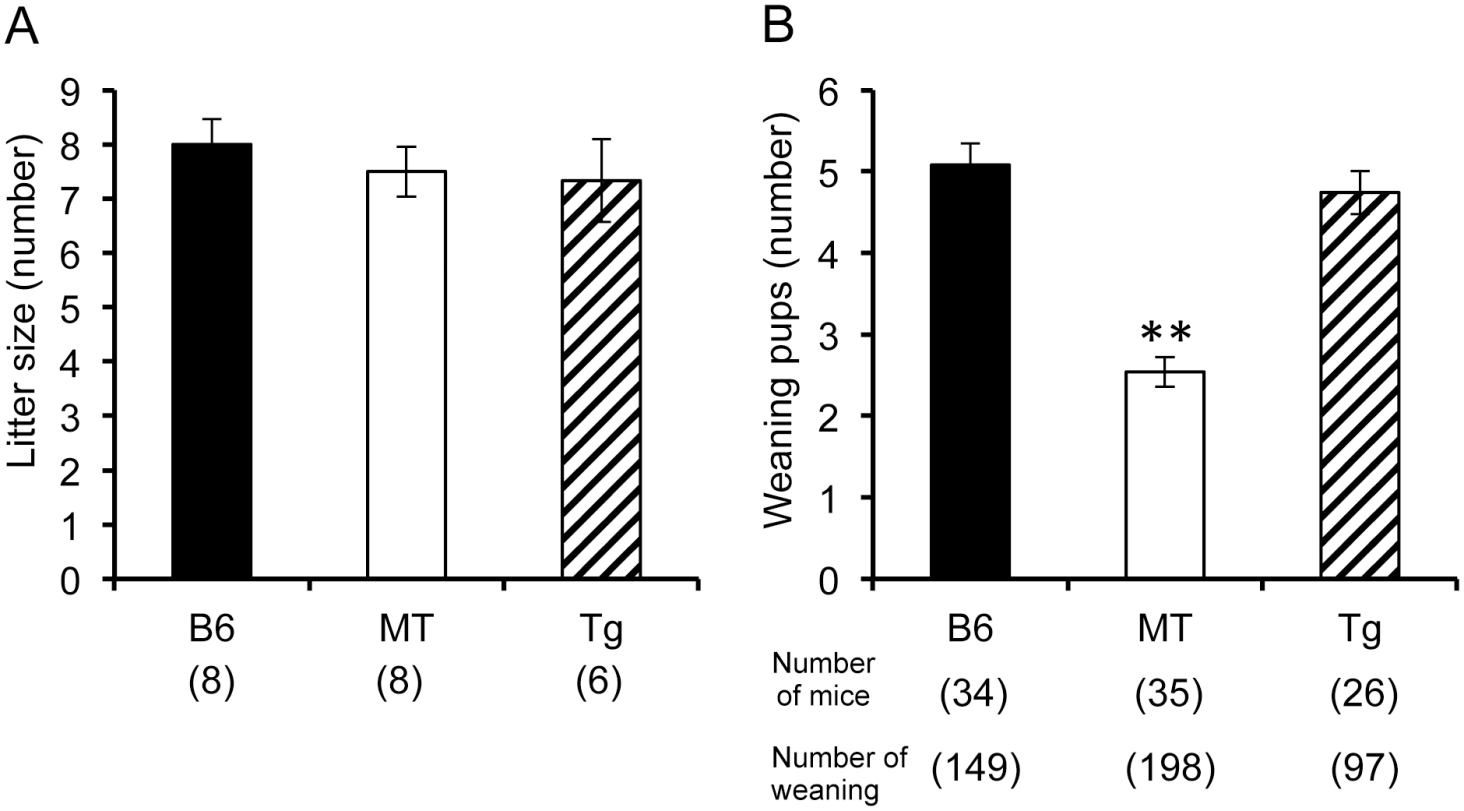
Effects of *Usp46* mutation on rate of weaning. A: Number of pups in the uterus prior to parturition. B: Number of pups weaned at approximately 25 days after birth. The number of mice is shown within parentheses. **p < 0.01, Kruskal-Wallis one-way ANOVA.

We counted the number of pups in the uterus just prior to parturition and found no significant difference between the 3 strains. However, at weaning, the number of pups in MT litters was significantly decreased compared to litters from B6 and *Usp46* transgenic mice (χ^2^ = 57.61, *p* < 0.01, Kruskal-Wallis one-way ANOVA, Fig 2).

### Maternal behavior

**Fig 3:**
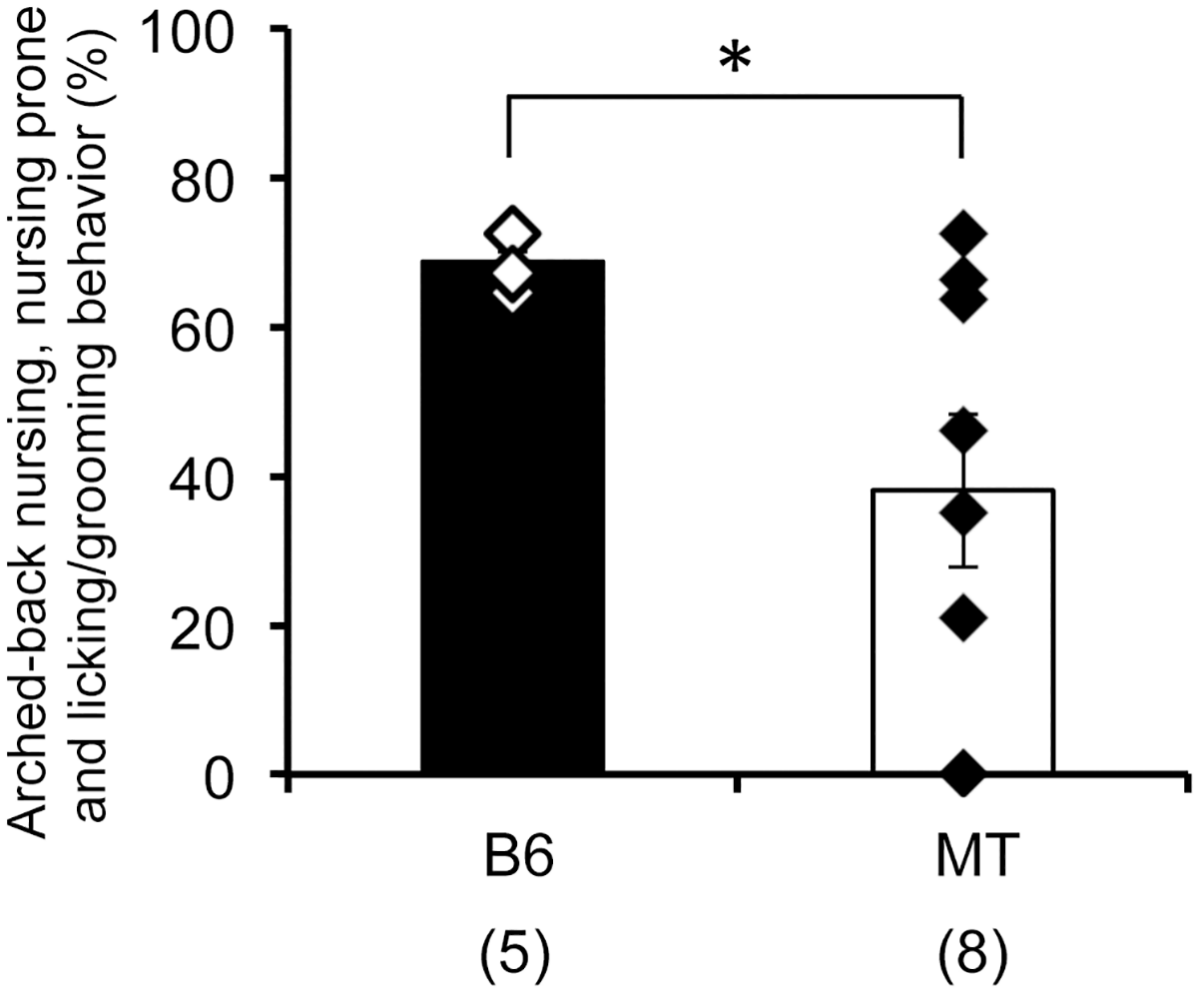
Maternal behaviors post parturition. Maternal behaviors (arched-back nursing, nursing in prone position and licking/grooming) were observed for 3 days following parturition. The ratio of occurrence of these behaviors is shown as the percent of total observation time. The number of mice used is shown within parentheses. *p < 0.05, Mann-Whitney U test.

Maternal behaviors could be categorized into arched-back nursing, nursing in a prone position and licking/grooming behavior. These behaviors were observed for 3 days following parturition. Although all B6 mice exhibited uniform duration of maternal behaviors and all B6 pups survived, MT mice showed large individual variations in maternal behaviors (Mann-Whitney U test, *p* < 0.05, Fig 3). MT mice whose whole litters died during these 3 days (5/8 litters) exhibited decreased duration of maternal behaviors compared to those whose litters survived.

### Fostering effects on maternal behavior in B6 mice

**Fig 4:**
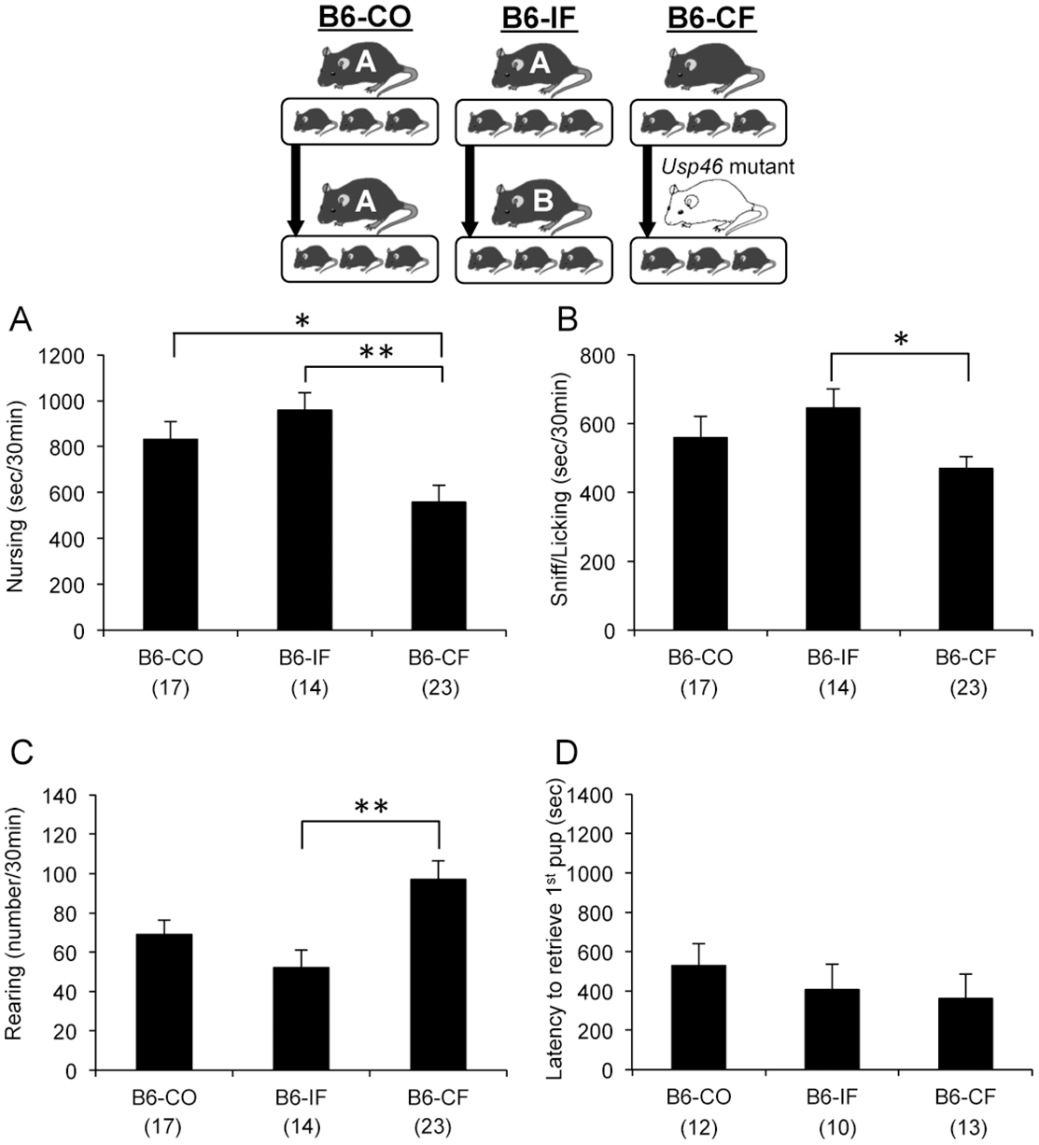
Fostering effects on maternal behavior in B6 mice. B6-CF exhibited a significant reduction in time spent nursing and sniffing/licking pup, and increased the number of instances of rearing. The number of mice used is shown within parentheses. *p < 0.05, **p < 0.01, one-way ANOVA with Tukey’s *post hoc* test.

We examined the effects of fostering by a non-biological mother and MT mother on maternal behavior in B6 mice (Fig 4). As observed in nursing mothers after parturition (Fig 3), MT mice showed significant impairments in maternal behavior compared to B6 mice (nursing: *F*_genetic effect (1,59)_ = 33.11, *p* < 0.01; sniffing/licking: *F*_genetic effect (1,59)_ = 7.61, *p* < 0.01; rearing: *F*_genetic effect (1,59)_ = 13.60, *p* < 0.01; latency to retrieve 1^st^ pup: *F*_genetic effect (1,41)_ = 7.27, *p* < 0.05; all two-way ANOVA) (S2 Fig). Although in-fostering effects were not observed, B6 mice fostered by MT mothers (B6-CF) exhibited a significant reduction in time spent nursing and sniffing/licking pup, and increased the number of instances of rearing (B6-CO vs. B6-CF, one-way ANOVA with Tukey’s *post hoc* test, *p* < 0.05; B6-IF vs. B6-CF, *p* < 0.01, *F*_(2, 51)_ = 7.30 for nursing, B6-IF vs. B6-CF, *p* < 0.05, *F*_(2, 51)_ = 3.10 for sniffing/licking, B6-IF vs. B6-CF, *p* < 0.01, *F*_(2, 51)_ = 6.49 for rearing) (Fig 4).

### Fostering effects on maternal behavior in MT mice

**Fig 5:**
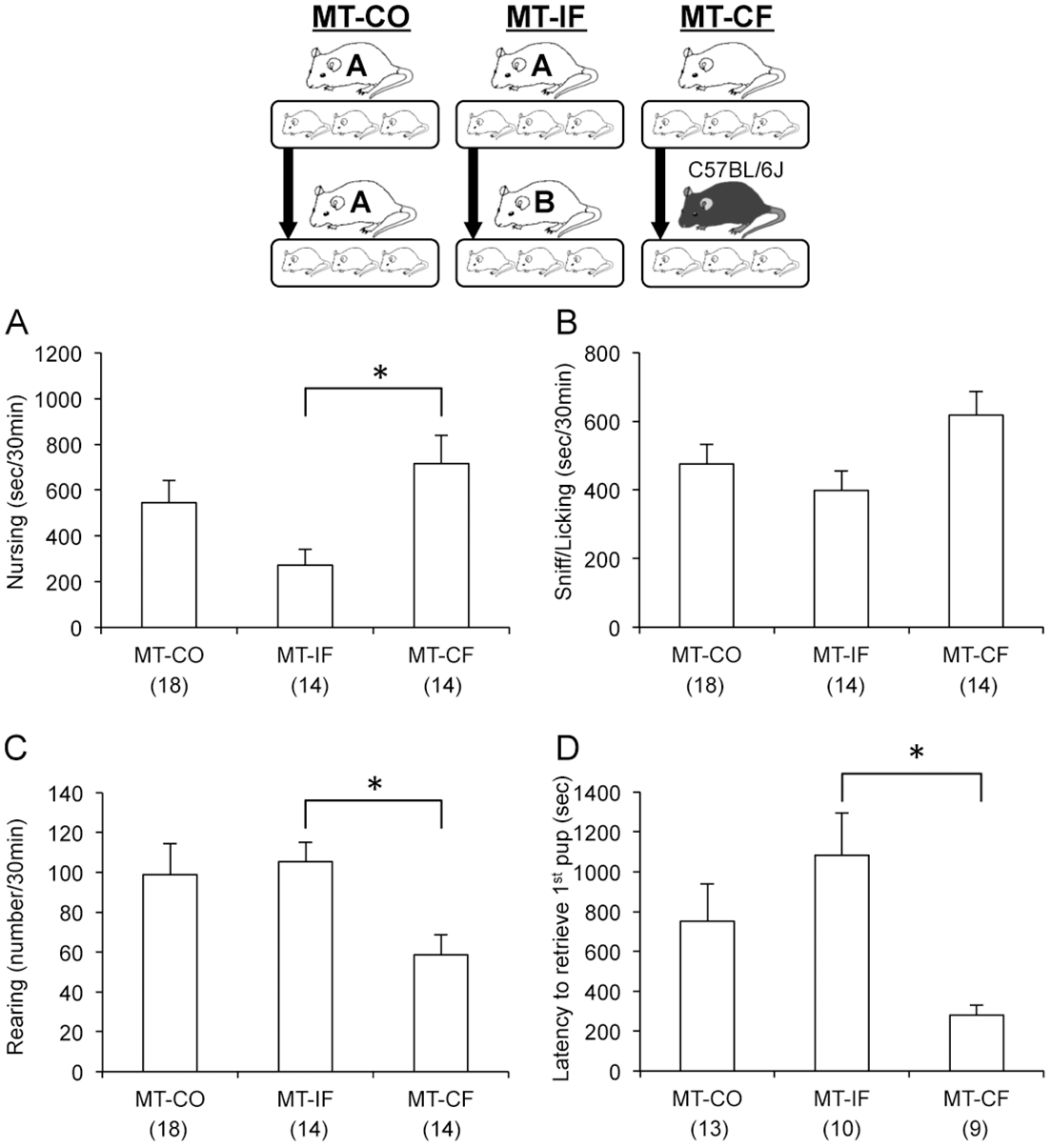
Fostering effects on maternal behavior in MT mice. MT-CF showed significant improvements in time spent nursing, rearing and latency to retrieve 1^st^ pup. The number of mice used is shown within parentheses. *p < 0.05, one-way ANOVA with Tukey’s *post hoc* test.

In fostering effects were not detected in MT mice as in B6 mice. However, MT mice fostered by B6 mothers (MT-CF) showed significant improvements in three parameters of maternal behaviors (MT-IF vs. MT-CF, one-way ANOVA with Tukey’s *post hoc* test, *p* < 0.05, *F*_(2, 43)_ = 4.60 for nursing, MT-IF vs. MT-CF, *p* < 0.05, *F*_(2, 43)_ = 3.71 for rearing, MT-IF vs. MT-CF, *p* < 0.05, *F*_(2, 29)_ = 4.61 for latency to retrieve 1^st^ pup) (Fig 5).

## Discussion

Maternal care is important for the physical and mental development of offspring. In particular, the early-life environment can significantly affect mental health in later life. Thus, prevention of child abuse has become an important public health concern. It is critical to understand the pathogenic mechanisms of child abuse and an appropriate animal model would be help in this endeavor. Thus, in this study, we evaluated the suitability of MT mice as a model for trans-generational transmission of maternal care that is critically involved in the pathological mechanisms of human child abuse. To develop an appropriate model, three criteria (face, construct and predictive validity), frequently applied to evaluate animal models of neuropsychiatric disorders, should be satisfied [20]. Child abuse has three prominent characteristics: transmission to subsequent generations; onset can be triggered by stressful environments; and there is recurrence within the same family [8–10, 21, 22]. In this study, we used cross-fostering experiments involving MT mice to investigate whether the maltreatment phenotype would be transmitted to the next generation, enabling assessment of one aspect of face validity.

The weaning rate of MT mice was significantly lower than B6 and *Usp46* transgenic mice; no significant differences were observed between B6 and *Usp46* transgenic mice. These results suggest that the *Usp46* mutation gives rise to the lower weaning rate. However, it is also possible that pups possessing the *Usp46* mutation are not strong enough to survive until weaning. Although this possibility cannot be excluded, the lower survival rate is likely due to poor maternal care during the early nursing period, as the survival rate of MT mice dramatically decreased within 3 days of parturition and was maintained at the same level afterwards (S3 Fig). It is known that following parturition, mice show a series of innate maternal behaviors, including placentophagia, licking, nursing, and pup-retrieval and grouping [23]. The dramatic change in surroundings mice encounter following parturition may have disturbed the expression of normal maternal behavior in MT mice. This speculation is supported by our previous results that show MT mice display neophobia-like behaviors [12]. Thus, it is likely that MT mice are vulnerable to stressful conditions. However, as shown in Fig 3, not all MT mice exhibited poor maternal behavior; normal maternal care, similar to that of B6 mice, was observed in some MT mice. This may be due to the fact that the *Usp46* mutation has moderate, not drastic, effects on behavioral phenotypes. Therefore, some MT mice that are rendered sensitive to stressful conditions may exhibit alterations in maternal care. If this were the case, MT mice would be a useful model for studying maltreating maternal behavior, as in most cases, this behavior occurs under excessive stress [22].

In this study, we evaluated whether maltreating maternal behaviors in MT mice are transmitted to the next generation by nongenomic means. To examine this, we performed an adoption study in which the biological offspring of B6 or MT mothers were cross-fostered to either B6 or MT dams. In most of previous studies, cross-fostering was conducted within 1 day of parturition [16, 24, 25]. In this study, however, offspring cross-fostered within 2 days after parturition were used, because several studies indicated that later adoption (e.g. postnatal day 7, 12) is effective to change behavioral phenotypes in the adult offspring [26–28]. First, we compared control and in-fostered groups for each strain, and showed no obvious effects in both strains (Figs 4 and 5). Next, we examined how postpartum maternal care shapes maternal behavior of grown female offspring. For B6 mice reared by MT mice, time spent nursing and sniffing/licking pup decreased, and the number of instances of rearing increased (Fig 4). On the other hand, in MT mice reared by B6 mice, most of maternal behaviors were improved (Fig 5). These results suggest that proper maternal behaviors can be shaped when adequate postpartum maternal care is given, even in genetically vulnerable mice. However, pup genotype must also contribute to the development of maternal behavior in adulthood, as MT mice fostered by non-biological MT mothers exhibited a significant reduction in time spent nursing and an increase in pup-retrieval latency when compared to B6 mice fostered by MT mothers (B6-CF vs. MT-IF). The deterioration of maternal behavior of MT-IF mice was also observed in the number of pups in the nest at the end of the experiment (S1 Table). Thus, both B6 and MT pups mothered by B6 mice showed similar maternal phenotypes once they became mothers (B6-IF vs. MT-CF). These data suggest that maternal care by B6 mothers is sufficient to inhibit the genetic vulnerability of MT mice.

From an evolutionally standpoint, maternal rejections which contribute to offspring independency can be interpreted by parent-offspring conflict arising from differences in optimal parental investment to an offspring [29]. In this theory, behavioral conflict between parents and offspring arise when parent investment terminates, but offspring demand as much as the investment given until that time. It is possible to consider that *Usp46* is involved in the adaptation of this behavior in the evolutionary process and impairment of the down-stream pathway of this gene affects the parent-offspring relationship.

We previously reported that *Usp46* regulates GABA_A_ receptor function [13]. Although a number of studies have demonstrated that the GABAergic system is implicated in regulation of maternal behavior [30–33], it has not been determined whether poor maternal behavior in MT mice is caused by dysfunction of GABA_A_ receptors. Further, the mechanism of nongenomic transmission of maternal care in these mice is unknown, although epigenetic programming by maternal care for subsequent maternal care has been well-described [2, 14–17, 34]. To elucidate these issues in these mice may lead to improved understanding of the mechanisms shaping maternal behavior, particularly because our attention in future work can be confined to down-stream pathways of *Usp46*.

## Supporting Information

S1 Fig Time sampling for behavioral observation. The ratio of occurrence of maternal behaviors was compared at three intervals (10 s observation at 2, 3 and 6 min intervals).
(TIF)

S2 Fig In-fostering effects on maternal behavior. MT mice (MT-CO and MT-IF) showed significantly poorer maternal behavior than B6 mice (B6-CO and B6-IF) across all parameters. *p < 0.05, **p < 0.01, two-way ANOVA.
(TIF)

S3 Fig Survival rate of B6 and MT mice during the nursing period. The survival rate of MT mice dramatically decreased within 3 days of parturition. The number of mice used is shown within parentheses. **p < 0.01, Student’s *t*-test.
(TIF)

S1 Table Results of pup-retrieval test. (PDF)

S2 Table The ARRIVE guidelines checklist. (PDF)
