# Supplementary figures and images for: Impaired Maternal Behavior in Usp46 Mutant Mice: A Model for Trans-Generational Transmission of Maternal Care
Source: PLoS One. 2015 Aug 18;10(8):e0136016. doi: 10.1371/journal.pone.0136016 (PMC4540444; doi:10.1371/journal.pone.0136016)

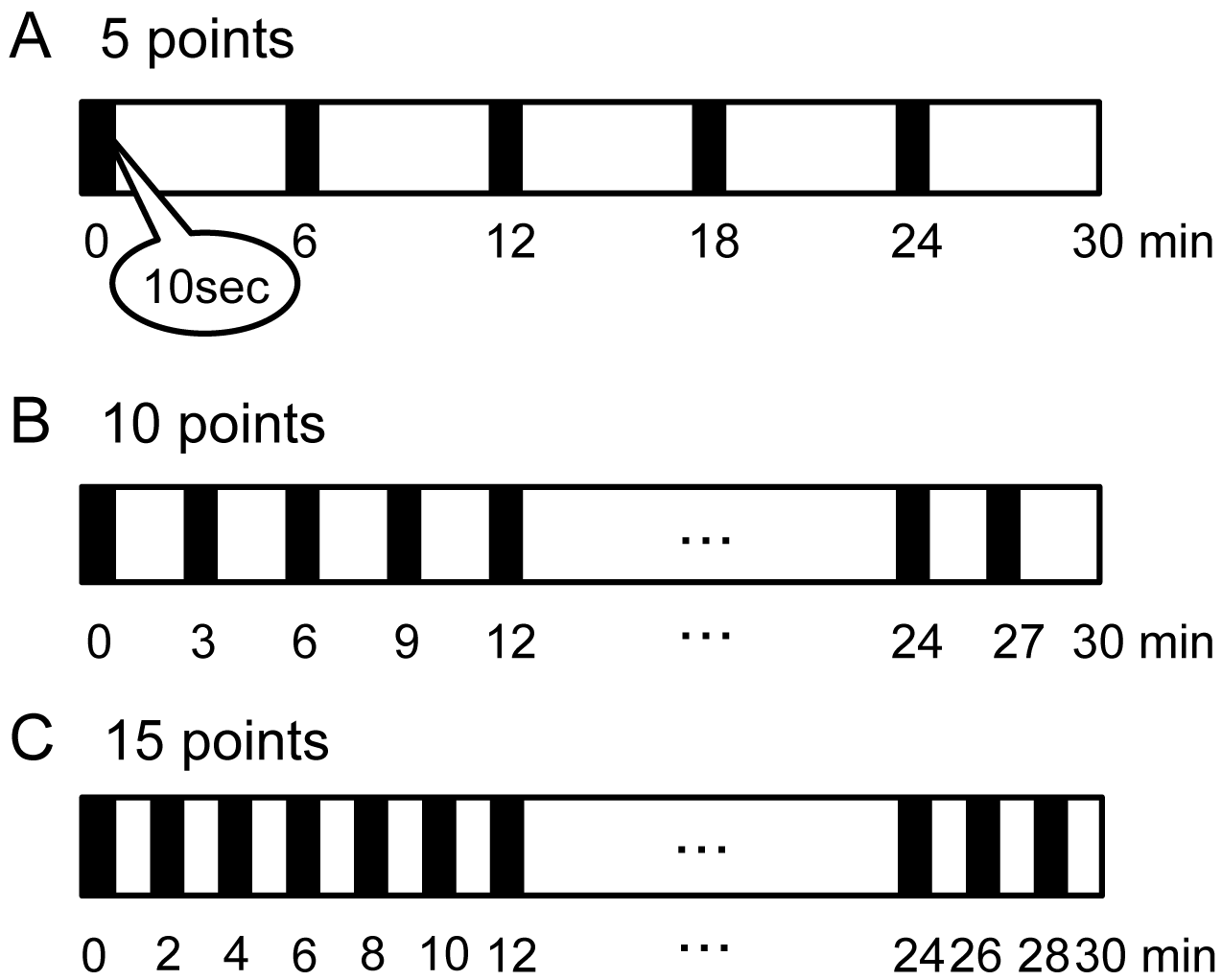

Supplement: S1 Fig — The ratio of occurrence of maternal behaviors was compared at three intervals (10 s observation at 2, 3 and 6 min intervals). (TIF) [file pone.0136016.s001.tif]

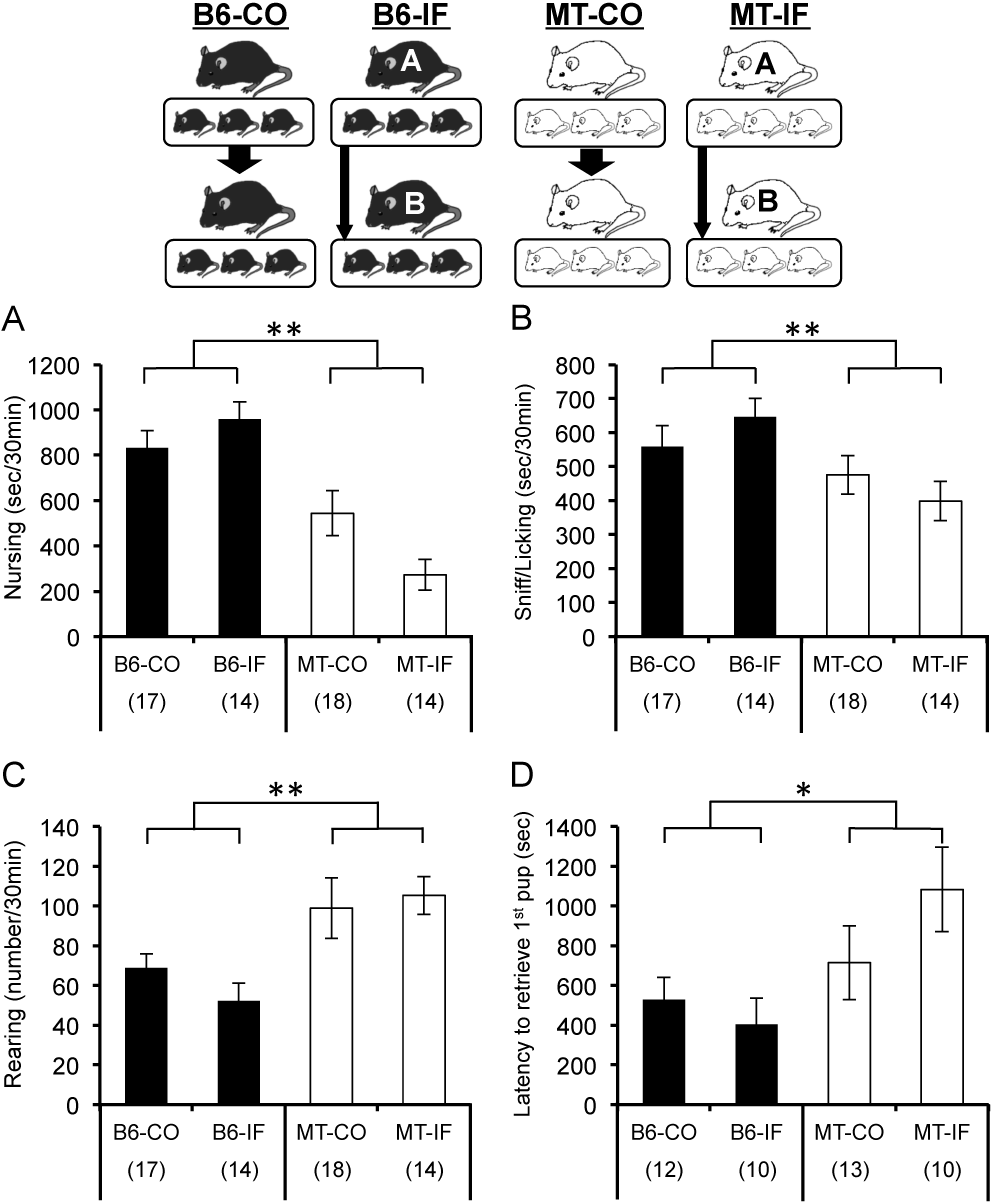

Supplement: S2 Fig — MT mice (MT-CO and MT-IF) showed significantly poorer maternal behavior than B6 mice (B6-CO and B6-IF) across all parameters. *p < 0.05, **p < 0.01, two-way ANOVA. (TIF) [file pone.0136016.s002.tif]

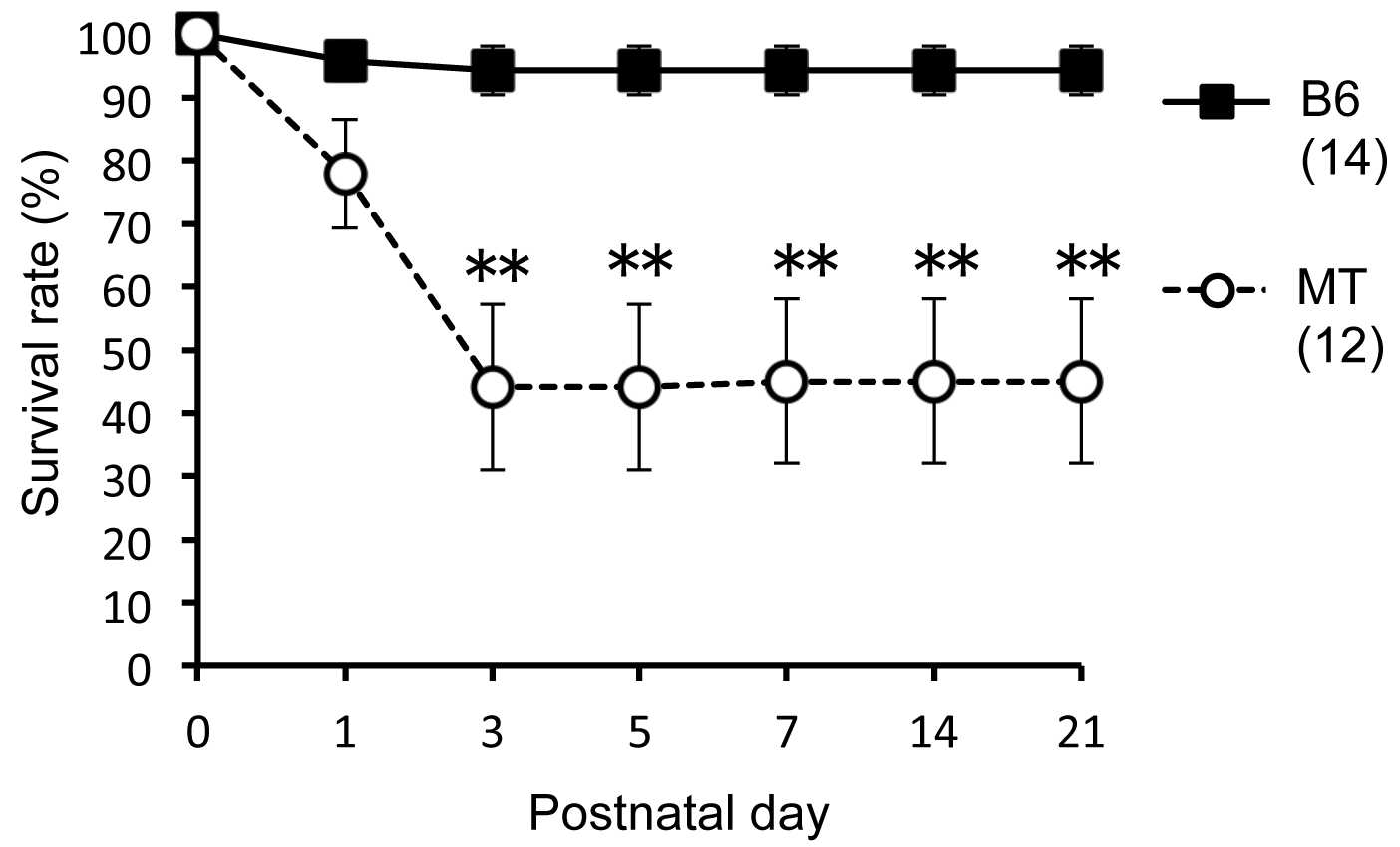

Supplement: S3 Fig — The survival rate of MT mice dramatically decreased within 3 days of parturition. The number of mice used is shown within parentheses. **p < 0.01, Student’s t-test. (TIF) [file pone.0136016.s003.tif]
