# Supplementary material for: Impaired Maternal Behavior in Usp46 Mutant Mice: A Model for Trans-Generational Transmission of Maternal Care
Source: PLoS One. 2015 Aug 18;10(8):e0136016. doi: 10.1371/journal.pone.0136016 (PMC4540444; doi:10.1371/journal.pone.0136016)
Supplement: S1 Table — (PDF) [file pone.0136016.s004.pdf]

Table S1. Results of pup-retrieval test

Pup retrieval test	Litter	Nursing (sec/30min)	Sniff/Licking (sec/30min)	Rearing (sec/30min)	Latency to retrieve 1st pup (sec)	2nd pup (sec)	3rd pup (sec)	Number of pups in a nest (number)	Infanticide
B6-CO (17)	5	832.0±78.2	558.3±62.1	68.9±7.2	529.4±110.7 (12)	769.6±135.4 (16)	883.0±161.3 (15)	2.7±0.2 (17)	0% (0/17)
B6-IF (14)	5	959.8±76.8	645.9±54.3	52.3±8.9	404.7±131.6 (10)	588.0±211.0 (9)	834.5±182.1 (12)	2.7±0.2 (14)	0% (0/14)
B6-CF (23)	5	557.2±74.4**	469.4±33.4	97.0±9.5	361.5±124.2** (13)	512.5±117.6** (18)	867.7±154.1** (17)	2.6±0.2* (23)	0% (0/23)
MT-CO (18)	5	543.7±99.3	475.7±57.4	99.0±15.3	751.2±187.0 (13)	851.8±175.3 (13)	1209.8±163.3 (16)	2.3±0.3 (18)	0% (0/18)
MT-IF (14)	5	272.0±68.3	398.1±57.5	105.4±9.7	1083.2±212.2 (10)	1269.7±167.5 (13)	1433.4±136.1 (13)	1.6±0.4 (14)	0% (0/14)
MT-CF (14)	5	716.1±122.0	617.6±69.8	58.8±9.9	279.0±51.3 (9)	591.5±117.4 (11)	978.1±191.3 (12)	2.5±0.2 (23)	0% (0/14)

Data are shown as the mean ± S.E.M. for the number of animals given in parentheses. ***p*< 0.01, **p*<0.05, vs. MT-IF, Student's *t*-test.
